# Leucyl-tRNA Synthetase Contributes to Muscle Weakness through Mammalian Target of Rapamycin Complex 1 Activation and Autophagy Suppression in a Mouse Model of Duchenne Muscular Dystrophy

**DOI:** 10.1016/j.ajpath.2024.04.006

**Published:** 2024-08

**Authors:** Jae-Sung You, Kate Karaman, Adriana Reyes-Ordoñez, Soohyun Lee, Yongdeok Kim, Rashid Bashir, Jie Chen

**Affiliations:** ∗ Department of Cell and Developmental Biology, University of Illinois at Urbana-Champaign, Urbana, Illinois; † Department of Bioengineering, University of Illinois at Urbana-Champaign, Urbana, Illinois; § Department of Materials Science and Engineering, University of Illinois at Urbana-Champaign, Urbana, Illinois; ¶ Department of Mechanical Science and Engineering, University of Illinois at Urbana-Champaign, Urbana, Illinois; ‡ Nick J. Holonyak Micro and Nanotechnology Laboratory, University of Illinois at Urbana-Champaign, Urbana, Illinois; ‖ Department of Biomedical and Translational Sciences, Carle Illinois College of Medicine, Urbana, Illinois

## Abstract

Duchenne muscular dystrophy (DMD), caused by loss-of-function mutations in the dystrophin gene, results in progressive muscle weakness and early fatality. Impaired autophagy is one of the cellular hallmarks of DMD, contributing to the disease progression. Molecular mechanisms underlying the inhibition of autophagy in DMD are not well understood. In the current study, the DMD mouse model *mdx* was used for the investigation of signaling pathways leading to suppression of autophagy. Mammalian target of rapamycin complex 1 (mTORC1) was hyperactive in the DMD muscles, accompanying muscle weakness and autophagy impairment. Surprisingly, Akt, a well-known upstream regulator of mTORC1, was not responsible for mTORC1 activation or the dystrophic muscle phenotypes. Instead, leucyl-tRNA synthetase (LeuRS) was overexpressed in *mdx* muscles compared with the wild type. LeuRS activates mTORC1 in a noncanonical mechanism that involves interaction with RagD, an activator of mTORC1. Disrupting LeuRS interaction with RagD by the small-molecule inhibitor BC-LI-0186 reduced mTORC1 activity, restored autophagy, and ameliorated myofiber damage in the *mdx* muscles. Furthermore, inhibition of LeuRS by BC-LI-0186 improved dystrophic muscle strength in an autophagy-dependent manner. Taken together, our findings uncovered a noncanonical function of the housekeeping protein LeuRS as a potential therapeutic target in the treatment of DMD.

Duchenne muscular dystrophy (DMD) is one of the most common forms of muscular dystrophies, caused by mutations in the X-linked dystrophin gene, with an estimated incidence of approximately 1 in 5000 live male births.^1^ Dystrophin deficiency results in progressive muscle weakness and deterioration of the muscular system, ultimately resulting in early death.^2^ Although gene therapies aimed at restoring dystrophin function are promising, the staggering diversity of the mutations and the heterogeneity of disease manifestation pose significant challenges.^3^ Therefore, identifying new molecular targets independent of dystrophin could greatly benefit therapeutic development. One of the cellular alterations underlying the DMD phenotypes is the impairment of autophagy.^4^, ^5^, ^6^, ^7^, ^8^, ^9^ Compromised muscle cell integrity in DMD generates defective organelles and toxic cellular constituents, which need to be removed through the autophagy process.^10^ However, in the DMD muscle, autophagy is severely repressed, exacerbating the disease.^4^, ^5^, ^6^, ^7^, ^8^ The molecular pathways that lead to the inhibition of autophagy in DMD are not well understood.

The dystrophin-deficient *mdx* mouse is the most widely used experimental model for DMD, harboring a nonsense point mutation in exon 23.^11^ *Mdx* mice enter an acute onset stage of its pathology at 3 to 4 weeks of age and show major DMD-like phenotypes, such as muscle injury and necrosis and, more important, muscle weakness.^12^^,^^13^ Although these phenotypes are compensated by robust muscle regeneration and repair in adult ages, a proteomics study has revealed that skeletal muscles of *mdx* mice exhibit highly similar protein signatures across all stages of dystrophic progression to that of *mdx53* mice,^14^ a model of more severe disease caused by a human DMD mutation.^14^ Therefore, the *mdx* mouse, especially at a young age, remains a good model for the studies of molecular mechanisms underlying DMD muscle pathology.

The Ser/Thr protein kinase mammalian (or mechanistic) target of rapamycin complex 1 (mTORC1), a master regulator of numerous cellular processes, including cell growth,^15^ is a major suppressor of autophagy.^16^, ^17^, ^18^ In the presence of nutrient/amino acid sufficiency and growth factor stimulation, active mTORC1 inhibits various steps of the autophagic process from initiation to autophagosome maturation, through direct phosphorylation of autophagy regulators, including unc-51–like kinase 1 (ULK1).^16^, ^17^, ^18^ mTORC1 activity is found to be elevated in human and mouse DMD skeletal muscles,^4^^,^^5^^,^^8^ and inhibiting mTORC1 by rapamycin or low-protein diet (amino acid deprivation) has shown improvements in dystrophic phenotypes, including autophagy restoration, in *mdx* mice.^4^, ^5^, ^6^^,^^19^^,^^20^ Tuberous sclerosis complex 2 (TSC2) inhibits mTORC1 by acting as a GTPase-activating protein for the small G protein Rheb, which directly activates mTORC1.^21^^,^^22^ In response to growth factor signals, the Ser/Thr kinase Akt is activated and subsequently phosphorylates and inhibits TSC2, thus activating mTORC1 via Rheb. One of the upstream activators of Akt is mTORC2, a mammalian target of rapamycin complex distinct from mTORC1 in composition and activity.^15^ Although Akt hyperactivity in *mdx* muscles has been reported,^4^^,^^5^^,^^23^^,^^24^ a direct connection between Akt and mTORC1 in autophagy regulation and DMD pathology has not been assessed.

Several upstream regulators and pathways mediate amino acid sensing and activation of mTORC1.^25^^,^^26^ Leucyl-tRNA synthetase (LeuRS) has a noncanonical function independent of protein synthesis by directly sensing the presence of leucine and regulating cell growth.^27^^,^^28^ Specifically, leucine-bound LeuRS interacts with the small G protein RagD and serves as its GTPase-activating protein.^27^ RagD in its GDP-bound form is an activator of mTORC1 on the lysosome in an amino acid–sensing pathway.^29^ A small-molecule chemical, BC-LI-0186, inhibits mTORC1 activation by disrupting the LeuRS-RagD interaction.^30^ The LeuRS-RagD-mTORC1 pathway plays a homeostatic role by suppressing injury-induced skeletal muscle regeneration in mice.^31^ However, this pathway has not previously been examined in DMD.

In the current study in *mdx* mice, activation of mTORC1, inhibition of autophagy, and the accompanying muscle weakness on loss of dystrophin were shown to be independent of Akt activation. Instead, LeuRS expression was elevated in the *mdx* muscle, and played a critical role in activating mTORC1 and suppressing autophagy. Remarkably, BC-LI-0186 inhibition of LeuRS and mTORC1 restored autophagy and rescued muscle strength in an autophagy-dependent manner in *mdx* muscles.

## Materials and Methods

### Animals

All animal experiments were performed in agreement with protocols approved by the Institutional Animal Care and Use Committee at the University of Illinois at Urbana-Champaign. *Mdx* mice [Research Resource Identifier (RRID): IMSR_JAX:001801] were originally purchased from the Jackson Laboratory (Bar Harbor, ME) and crossed with the authors’ in-house C57BL/6N mice. Heterogeneous X^mdx^:X^+^ female mice and wild-type male mice were then bred to obtain *mdx* and wild-type (WT) male offspring. Genotyping for dystrophin was performed as previously described.^32^ Mice of the same age, between 4 and 13 weeks old, were randomly assigned to the various experimental groups and anesthetized with isoflurane during all surgical procedures and at the end of the experiments for euthanasia. Animals were housed in cages connected to an EcoFlo ventilation system (Allentown Inc., Allentown, NJ) in a room kept at 23°C with a 12-hour light/dark cycle and received a pellet diet and water *ad libitum*.

### Drug Treatments

All drugs were diluted in phosphate-buffered saline (PBS) and injected intraperitoneally into mice. The injections were repeated every 24 hours at the following final concentrations (mg drug/kg body weight) unless otherwise noted: chloroquine diphosphate (stock in water; Sigma-Aldrich, St. Louis, MO) at 50 mg/kg, triciribine (stock in dimethyl sulfoxide; Sigma-Aldrich) at 2 mg/kg, and BC-LI-0186 (stock in dimethyl sulfoxide; Sigma-Aldrich) at 5 mg/kg. For acute treatment, drugs were injected one time as follows: insulin at 5 U/kg 30 minutes before sample collection and BC-LI-0186 at 20 mg/kg 4 hours before sample collection. Control mice were injected with an equivalent volume of vehicle diluted in PBS.

### Muscle Force Measurement

Muscle force measurement in tibialis anterior (TA) muscle was performed *in situ* using a 1300A Whole-Animal System (Aurora Scientific, Aurora, ON, Canada), as described previously.^33^ Briefly, the mouse was stabilized on an isothermal stage set at 38°C by inserting a needle through a fixed post and patella tendon. The distal tendon of the TA muscle was connected to the lever arm of the force transducer through a 3 to 0 suture line. The muscle was directly stimulated through two electrodes with 0.2-m square-wave pulses at 0.2 mA and adjusted to optimal muscle length where maximal twitch force was produced. The maximum isometric tetanic force was determined in the frequency range of 50 to 200 Hz with a 300-m pulse duration, with each contraction separated by a 1-minute rest. Specific isometric tetanic force was then calculated by dividing maximal isometric tetanic force by physiological cross-sectional area [muscle mass/(fiber length × muscle density 1.06 g/cm^3^)].

### Antibodies

Antibodies against the following proteins were from Cell Signaling Technology (Danvers, MA): glyceraldehyde-3-phosphate dehydrogenase (GAPDH; catalog number 2118, RRID: AB_561053), phosphorylated Ser473 (pSer473)–Akt (catalog number 9271, RRID: AB_329825), Akt (catalog number 9272, RRID: AB_329827), phosphorylated Thr1465–TSC2 (catalog number 3617, RRID: AB_490956), TSC2 (catalog number 4308, RRID: AB_10547134), pSer9–glycogen synthase kinase 3 β (GSK3β) (catalog number 5558, RRID: AB_10013750), GSK3β (catalog number 9832, RRID: AB_10839406), pSer252–forkhead box O3a (FoxO3a) (catalog number 9466, RRID: AB_2106674), FoxO3 (catalog number 2497, RRID: AB_836876), pSer240/244-S6 (catalog number 5364, RRID: AB_10694233), S6 (catalog number 2217, RRID: AB_331355), pSer757-ULK1 (catalog number 14202, RRID: AB_2665508), ULK1 (catalog number 8054, RRID: AB_11178668), microtubule-associated protein 1A/1B-light chain 3 (LC3A/B) (catalog number 4108, RRID: AB_2137703), and p62 (catalog number 23214, RRID: AB_2798858). Anti-LeuRS was a gift from Dr. Susan Martinis (University of Illinois at Urbana-Champaign).^34^ Anti–glutamyl-prolyl-tRNA synthetase (EPRS) (catalog number ab31531, RRID: AB_880047) was from Abcam (Cambridge, UK). Anti–glycyl-tRNA synthetase (GlyRS) (catalog number LS-B9126-200) was from LSBio (Lynnwood, WA). Anti–tyrosyl-tRNA synthetase (TyrRS) (catalog number HPA018954, RRID: AB_1858915) and anti-laminin (catalog number L9393, RRID:AB_477163) were from Sigma-Aldrich. Peroxidase-conjugated anti-rabbit IgG (catalog number 111-036-003, RRID: AB_2337942) and anti-mouse IgG (catalog number 115-036-003, RRID: AB_2338518) were from Jackson Immuno Research Laboratories (West Grove, PA). Alexa Fluor 488–conjugated anti-mouse IgG (catalog number A-11001, RRID: AB_2534069) and Alexa Fluor 594–conjugated anti-rabbit IgG (catalog number A-11012, RRID: AB_2534079) were from Thermo Fisher Scientific (Waltham, MA).

### Western Blot Analysis

On collection, TA muscles were snap frozen in liquid nitrogen. Frozen TA muscle tissues were homogenized with a Polytron in ice-cold buffer containing 20 mmol/L Tris (pH 7.4), 0.3% Triton X-100, 2 mmol/L EGTA, 2 mmol/L EDTA, 0.1 mmol/L Na_3_VO_4_, 25 mmol/L NaF, 25 mmol/L β-glycerophosphate, and 1× protease inhibitor cocktail (P8240; MilliporeSigma, Burlington, MA). Protein concentrations of the homogenates were measured with the DC protein assay kit (Bio-Rad, Hercules, CA). An equal amount of protein from each sample was boiled in Laemmli buffer, resolved on SDS-PAGE, transferred onto polyvinylidene difluoride membrane (MilliporeSigma), blocked with 5% dry milk in PBS with 0.5% Tween-20, and incubated with the appropriate antibodies per manufacturers' recommendations. After washing in PBS with 0.5% Tween-20, blots were developed using the SuperSignal West Pico PLUS Chemiluminescent Substrate (Thermo Fisher Scientific) and visualized on an iBright CL1000 Imaging System (Thermo Fisher Scientific). Quantification by densitometry was performed using ImageJ version 1.53K (NIH, Bethesda, MD: *https://imagej.nih.gov/ij*).

### Quantitative RT-PCR

On collection, TA muscles were snap frozen in liquid nitrogen. Frozen tissues were homogenized with a ribonuclease-free pestle in ice-cold TRIzol (Invitrogen, Waltham, MA). Total RNA was extracted and reverse transcribed using the GeneJET RNA Purification Kit (Thermo Fisher Scientific) and the qScript cDNA Synthesis Kit (Quanta Bioscience, Beverly, MA), respectively. cDNA was subjected to real-time quantitative PCR on a StepOnePlus Real-Time PCR System (Applied Biosystems, Waltham, MA) using SYBR Green. *Gapdh* was used as a reference to obtain the relative fold change for target samples using the comparative C_T_ method. The primer sequences used are as follows: *Gapdh*, 5′-GCAGCCTCGTCCCGTAGAC-3′ (forward) and 5′-ATGGCAACAATCTCCACTTTGC-3′ (reverse); *Lars*, 5′-GCCGAGAGTCTTGGATTTAGAG-3′ (forward) and 5′-CCAGCTCGAGAGAATTGGTTAG-3′ (reverse); *Cd74*, 5′-CCTCAAGGAAGAAGAACCCAAG-3′ (forward) and 5′-GCTGAGCAAGGAACCTGAAA-3′ (reverse); *Adgre1*, 5′-ACAGTCATCTCCCTGGTATGT-3′ (forward) and 5′-TGCAGGTGCATGTAGGTATTG-3′ (reverse); *Nos2*, 5′-CTTGGAGCGAGTTGTGGATT-3′ (forward) and 5′-CTCTTGTCTTTGACCCAGTAGC-3′ (reverse); *Tnf*, 5′-GGGAGAACAGAAACTCCAGAAC-3′ (forward) and 5′-CAGTGAGTGAAAGGGACAGAAC-3′ (reverse); *Il6*, 5′-CTTCCATCCAGTTGCCTTCTT-3′ (forward) and 5′-ATCCTCTGTGAAGTCTCCTCTC-3′ (reverse); *Gabarapl1*, 5′-CATCGTGGAGAAGGCTCCTA-3′ (forward) and 5′-ATACAGCTGGCCC ATGGTAG-3′ (reverse); *Bnip3*, 5′-TTCCACTAGCACCTTCTGATGA-3′ (forward) and 5′-GAACACGCATTTACAGAACAA-3′ (reverse); *Uvrag*, 5′-GACCACGAGACAGTTGAGATAG-3′ (forward) and 5′-GCAGGGACAATGGACTTAGAA-3′ (reverse); and *Atp6v0c*, 5′-CCCTAGAGTGCTCCTGTGTATAA-3′ (forward) and 5′-GCTCCACAGACGCATGAATAG-3′ (reverse).

### Immunohistochemistry

On collection, TA muscles were submerged in OCT compound (Tissue-Tek; Sakura Finetek, Alphen aan den Rijn, the Netherlands) and frozen in liquid nitrogen–chilled isopentane. The muscle in OCT compound was cut crosswise at 10-μm thickness at midbelly with a Microm HM550 cryostat (Thermo Fisher Scientific). The cross-sections were placed on microscope slides, fixed in acetone for 10 minutes at −30°C, rehydrated with PBS for 15 minutes, incubated in solution A (0.5% bovine serum albumin and 0.5% Triton X-100 in PBS) for 20 minutes, and probed with rabbit anti-laminin antibody in solution A for 1 hour. After washing with PBS, the sections were incubated with fluorophore-conjugated anti-mouse or anti-rabbit IgG secondary antibodies in solution A for 1 hour. After a final wash with PBS, fluorescent images were viewed using a Leica DMI 4000B microscope (Leica, Wetzlar, Germany) with a Fluotar 4× objective and captured with a Retiga EXi camera (QImaging, Surrey, BC, Canada) equipped with Image Pro Express software version 11 (Media Cybernetics, Rockville, MD). All procedures were performed by investigators blinded to sample identification.

### Statistical Analysis

All data are presented as means ± SEM, with individual data points shown in graphs (the number of data points represents *n*). The sample size for each experiment was determined on the basis of previous relevant publications^31^^,^^33^ and preliminary data. A quantified sample value that deviated more than three times SD from the mean in a given group was removed as an outlier. Statistical significance was determined by two-tailed unpaired *t*-tests or two-way analysis of variance followed by the Student-Newman-Keuls *post hoc* test, as specified in figure legends. All statistical analyses, including assumption tests, were performed using SigmaPlot 14.0 (Grafiti LLC, Palo Alto, CA).

## Results

### Akt Is Not Involved in Autophagy Impairment and Muscle Weakness in *mdx* Mice

**Figure 1:**
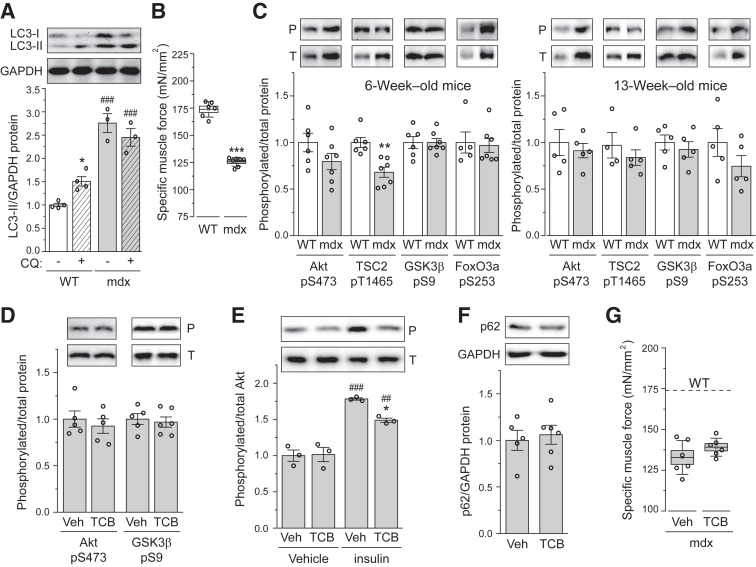
Akt is not involved in autophagy impairment and weakness in *mdx* muscle. **A** and **B:** Tibialis anterior (TA) muscles from 6-week–old wild-type (WT) and *mdx* mice treated with chloroquine (+CQ) or vehicle (−CQ) for 7 days were analyzed for protein expression of LC3 isoforms and glyceraldehyde-3-phosphate dehydrogenase (GAPDH; loading control) by Western blot analysis (**A**) or specific isometric tetanic force (**B**). **C:** TA muscles from 6-week–old (**left panel**) and 13-week–old (**right panel**) WT and *mdx* mice were analyzed for phosphorylation of Akt and Akt substrates by Western blot analysis. Results of quantification are shown as the relative ratio of phosphorylated (P)/total (T) protein, with representative blots shown. **D:** TA muscles from 6-week–old mdx mice treated with triciribine (TCB) or vehicle (Veh) for 7 days were analyzed for phosphorylation of Akt and GSK3β as in **C**. **E:** Six-week–old *mdx* mice were treated with triciribine (24 hours) and insulin (30 minutes) before sample collection. TA muscles were analyzed for phosphorylation of Akt as in **C**. **F** and **G:** TA muscles treated as in **D** were analyzed for protein expression of p62 and GAPDH (**F**) or specific isometric tetanic force (**G**). Data points representing individual mice are shown. **A**–**G:** Statistical analysis was performed using two-way analysis of variance (**A** and **E**) or unpaired *t*-test (**B**–**D**, **F**, and **G**). Data are presented as means ± SEM (**A** and **C**–**F**) or means ± SEM (box) and SD (whisker; **B** and **G**). ∗*P* < 0.05, ∗∗∗*P* < 0.001 for the effect of CQ or *mdx*; ^##^*P* < 0.01, ^###^*P* < 0.001 for the effect of *mdx* or insulin.

Consistent with reports in the literature,^4^, ^5^, ^6^, ^7^, ^8^ increased accumulation of LC3-II in TA muscles of *mdx* mice was observed, which was not further increased by chloroquine (CQ) treatment (Figure 1A), suggesting that autophagy flux was impaired in the *mdx* muscle. Specific muscle force was also significantly reduced in *mdx* muscles compared with WT (Figure 1B). To identify potential upstream regulators responsible for the impaired autophagy, Akt activation was examined. Western blot analysis of muscle homogenates was performed for TA of 6- and 13-week–old *mdx* mice in comparison to age-matched WT mice. No significant difference in specific activity of Akt was found, as reflected by the ratio of pSer473-Akt and total Akt (referred to as pAkt herein), between *mdx* and WT at either age (Figure 1C). Also, no difference was detected comparing *mdx* and WT muscles in the phosphorylation of three direct substrates of Akt, TSC2, GSK3β, and FoxO3a, except that at 6 weeks of age phosphorylation of TSC2 in *mdx* muscles was significantly lower than that in WT muscles (Figure 1C).

To assess the role of Akt in the inhibition of autophagy in the *mdx* muscle, mice were treated systemically with the Akt inhibitor triciribine (TCB), which blocks Akt membrane recruitment and activation.^35^ At a dose twice as high as was sufficient to inhibit Akt activation in ARHGEF3 (upstream inhibitor of Akt)–deficient muscle after injury,^33^ TCB did not affect the basal level of Akt activity or phosphorylation of GSK3β (Figure 1D). On the other hand, insulin-stimulated Akt phosphorylation was significantly dampened by TCB (Figure 1E), confirming the inhibitor efficacy. Neither autophagy, as measured by p62 levels (Figure 1F), nor specific muscle force (Figure 1G) in *mdx* was affected by TCB. Hence, Akt does not appear to contribute significantly to autophagy impairment or muscle weakness in dystrophic muscles of *mdx* mice.

### LeuRS Is Responsible for Hyperactivity of mTORC1 in the *mdx* Muscle

**Figure 2:**
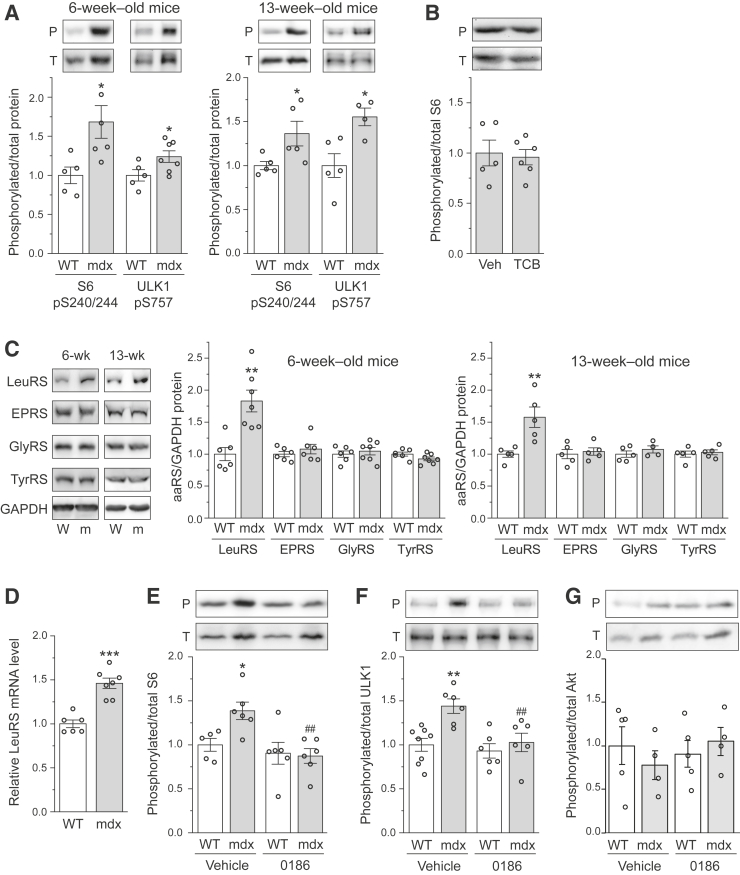
Leucyl-tRNA synthetase (LeuRS) is responsible for hyperactivity of mammalian target of rapamycin complex 1 in the *mdx* muscle. **A:** Tibialis anterior (TA) muscles from 6-week–old (**left panel**) and 13-week–old (**right panel**) wild-type (WT) and *mdx* mice were analyzed for phosphorylation of S6 and ULK1 by Western blot analysis. Results of quantification are shown as the relative ratio of phosphorylated (P)/total (T) protein, with a representative set of blots shown. **B:** TA muscles from 6-week–old *mdx* mice treated with triciribine (TCB) or vehicle (Veh) for 14 days were analyzed for phosphorylation of S6 as in **A**. **C:** TA muscles from 6- and 13-week–old WT (W) and *mdx* (m) mice were analyzed for protein expression of various aminoacyl-tRNA synthetases (aaRSs). Results of quantification are shown as the relative ratio of aaRS/glyceraldehyde-3-phosphate dehydrogenase (GAPDH; loading control), with representative blots shown at the left. **D:** TA muscles from 6-week–old WT and *mdx* mice were analyzed for LeuRS mRNA expression by quantitative RT-PCR. **E**–**G:** TA muscles from 6-week–old *mdx* mice treated with an acute dose of BC-LI-0186 (0186) or vehicle were analyzed for phosphorylation of S6 (**E**), ULK1 (**F**), and Akt (**G**) as in **A**. Data points representing individual mice are shown. **A**–**G:** Statistical analysis was performed using two-way analysis of variance (**E**–**G**) or unpaired *t*-test (**A**–**D**). Data are presented as means ± SEM (**A**–**G**). ∗*P* < 0.05, ∗∗*P* < 0.01, and ∗∗∗*P* < 0.001 for the effect of *mdx*; ^##^*P* < 0.01 for the effect of 0186.

Next, mTORC1 activation was examined by measuring the phosphorylation of S6 (pS6), a substrate of S6 kinase 1, which is a direct target of mTORC1. As shown in Figure 2A, pS6 was significantly elevated in *mdx* muscles compared with WT, in animals at both 6 and 13 weeks of age. Rapamcin administration nearly abolished the phosphorylation of S6 in both WT and *mdx* muscles (Supplemental Figure S1), confirming that pS6 faithfully represented the activity of S6 kinase 1 under the current experimental conditions. The mTORC1 substrate ULK1, which regulates autophagy, was also hyper-phosphorylated in *mdx* muscles compared with WT (Figure 2A), consistent with the role of mTORC1 in suppressing autophagy in the dystrophic muscles. The Akt inhibitor TCB did not affect pS6 (Figure 2B), suggesting that Akt is not a major contributor to the activation of mTORC1 in *mdx* muscles.

With Akt uncoupled from mTORC1 activation, LeuRS, another regulator upstream of mTORC1, was studied. Interestingly, the protein level of LeuRS was markedly elevated in the *mdx* muscle at both 6 and 13 weeks of age, whereas several other aminoacyl-tRNA synthetases were expressed at comparable levels in WT and *mdx* muscles (Figure 2C). The mRNA of LeuRS was also expressed at a higher level in *mdx* (Figure 2D). Next, the animals were treated with BC-LI-0186 (also referred to as 0186 hereafter), a specific inhibitor of LeuRS-RagD interaction^30^ upstream of mTORC1. The increased levels of pS6 and phosphorylated ULK1 in *mdx* muscles were abolished by 0186 (Figure 2, E and F), whereas pAkt was not affected by the drug (Figure 2G). These results suggest that LeuRS, with its elevated expression, is a significant contributor to the activation of mTORC1 observed in *mdx* muscles.

### BC-LI-0186 Restores Autophagy and Ameliorates Damage in Dystrophic Muscles

**Figure 3:**
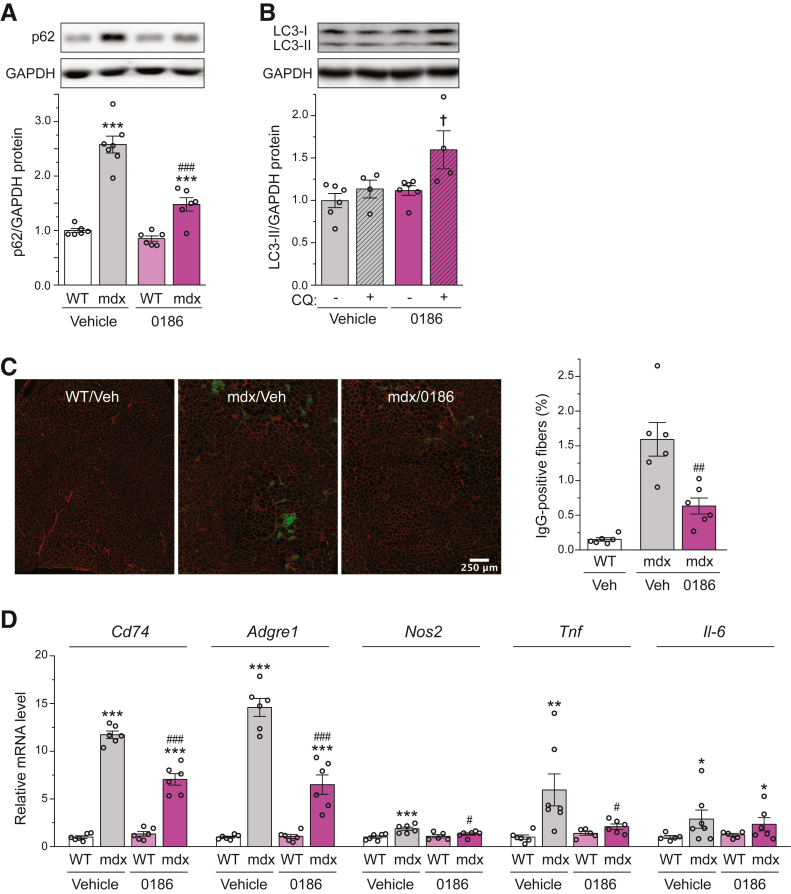
BC-LI-0186 (0186) restores autophagy and ameliorates damage in dystrophic muscles. **A:** Tibialis anterior (TA) muscles from 6-week–old wild-type (WT) and *mdx* mice treated with 0186 or vehicle (Veh) for 14 days were analyzed for protein expression of p62 and glyceraldehyde-3-phosphate dehydrogenase (GAPDH; loading control) by Western blot analysis. Results of quantification are shown as the relative ratio of p62/GAPDH, with representative blots shown. **B:** TA muscles from 6-week–old *mdx* mice treated with BC-LI-0186 or vehicle for 14 days with or without chloroquine (CQ) were analyzed for protein expression of LC3-II and GAPDH as in **A**. **C** and **D:** TA muscles treated as in **A** were analyzed for the percentage of IgG-positive myofibers by immunostaining (**C**) or relative mRNA expression of various inflammatory genes by quantitative RT-PCR (**D**). Data points representing individual mice are shown. **A**–**D:** Statistical analysis was performed using two-way analysis of variance (**A**, **B**, and **D**) or unpaired *t*-test (**C**). Data are presented as means ± SEM (**A**–**D**). ∗*P* < 0.05, ∗∗*P* < 0.01, and ∗∗∗*P* < 0.01 for the effect of *mdx*; ^#^*P* < 0.05, ^##^*P* < 0.01, and ^###^*P* < 0.001 for the effect of 0186; ^†^*P* < 0.05 for the effect of CQ. Scale bar = 250 μm (**C**).

Increased accumulation of p62 in the *mdx* muscle compared with WT was observed (Figure 3A), indicating blocked autophagy. Treatment of *mdx* mice with 0186 significantly reduced p62 levels in the muscles, whereas the drug had no effect in WT muscles (Figure 3A). In addition, 0186 increased LC3-II levels in the presence of CQ (Figure 3B). These observations suggest that inhibition of LeuRS-RagD-mTORC1 signaling restored autophagy in dystrophic muscles. The mRNA expression of several autophagy genes was also examined, including *Atp6v0c*, *Bnip3*, *Gabarapl1*, and *Uvrag*. These genes were differentially expressed in *mdx* muscles compared with the WT, but 0186 treatment had minimal effects on the changes (Supplemental Figure S2), consistent with a direct role of mTORC1-ULK1 signaling rather than autophagy gene expression in the observed restoration of autophagy by 0186 treatment.

The restoration of autophagy by 0186 coincided with a reduced number of IgG-infiltrated necrotic fibers in the *mdx* muscles, reflecting attenuated muscle damage (Figure 3C). As DMD muscle fibers go through constant cycles of degeneration and regeneration, inflammatory cells infiltrate and further exacerbate the disease progression of DMD.^36^ Less severe muscle damage would be expected to result in dampened inflammatory response. Indeed, it was observed that 0186 treatment significantly reduced the expression of several inflammatory genes in the *mdx* muscles, including *Cd74*, *Adgre1*, *Nos2*, and *Tnf* (Figure 3D). The authors’ observation is consistent with the reported rapamycin effect on reducing the number of infiltrating immune cells in *mdx* muscles.^20^ The effect of rapamycin, and most likely also of 0186, may be the consequence of reduced muscle damage, direct immunosuppression, or both, as rapamycin is a well-known immunosuppressant.^37^

### BC-LI-0186 Rescues Muscle Strength via Autophagy

**Figure 4:**
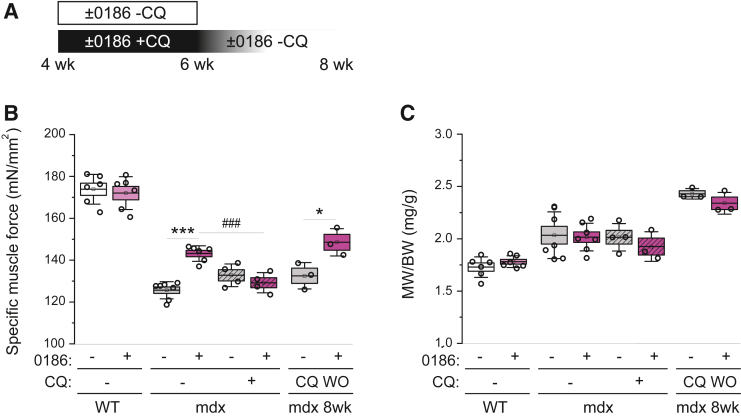
BC-LI-0186 (0186) rescues muscle strength via autophagy. **A:** Experimental design: 4-week–old wild-type (WT) and *mdx* mice were treated with 0186 or vehicle for 2 weeks in the presence or absence of chloroquine (CQ), with some mice followed by additional treatment of BC-LI-0186 or vehicle for 2 weeks in the absence of CQ [CQ washout (WO)]. **B:** After the treatments described in **A**, tibialis anterior (TA) muscles were subjected to specific isometric tetanic force measurement. **C:** After the treatments described in **A**, TA muscles were isolated and analyzed for the ratio of muscle weight (MW; mg)/body weight (BW; g). Data points representing individual mice are shown. Statistical analysis was performed using two-way analysis of variance, except for the *mdx* 8 weeks data analyzed using unpaired *t*-test. Data are presented as means ± SEM (box) and SD (whisker; **B** and **C**). ∗*P* < 0.05, ∗∗∗*P* < 0.01; ^###^*P* < 0.001.

Finally, the effect of 0186 on *mdx* muscle function was examined by measuring force generation, following the treatment schedule shown in Figure 4A. Again, specific muscle force was significantly lower in *mdx* mice compared with WT mice at 6 weeks of age (Figure 4B). Two weeks of BC-LI-0186 treatment had no effect on WT muscle force, but it partially and significantly rescued force generation in *mdx* mice. Blocking autophagic flux by administering CQ to *mdx* mice abolished the enhancement of specific muscle force by 0186 (Figure 4B). Furthermore, when the animals were allowed to recover from the CQ treatment for 2 weeks (CQ washout), the force-promoting effect of 0186 was restored (Figure 4B). Neither 0186 nor CQ affected muscle mass in WT or *mdx* mice (Figure 4C). Taken together, these observations strongly suggest that 0186 ameliorates muscle weakness in *mdx* mice by restoring autophagy.

## Discussion

Impaired autophagy is a well-documented cellular phenotype of DMD, and understanding the underlying molecular mechanisms is key to therapeutic explorations. The current study revealed elevated expression of LeuRS in the skeletal muscle of *mdx* mice and its pathologic role in the hyperactivation of mTORC1, suppression of autophagy, and muscle damage and weakness. Inhibiting mTORC1 activity and restoring autophagy using a specific inhibitor of the LeuRS-RagD-mTORC1 pathway led to improved muscle strength, accompanied by reduced muscle damage and inflammation. These observations reaffirm the importance of autophagy as a therapeutic target in DMD and uncover the therapeutic potential of targeting LeuRS.

The elevated LeuRS expression alone may not be sufficient for the observed hyperactivation of mTORC1 in dystrophic muscles, because leucine binding to LeuRS is necessary for the activation of Rag and subsequently mTORC1.^27^ Interestingly, Quy et al^38^ reported that autophagy is suppressed in denervated mouse muscles through constitutive activation of mTORC1 in a proteasome-dependent manner. Specifically, protein degradation results in elevated cellular amino acid levels in denervated muscles, which are likely responsible for the activation of mTORC1.^38^ It is plausible that increased protein degradation in dystrophic muscles can supply excess leucine, the necessary cofactor to permit the elevated LeuRS to hyperactivate RagD-mTORC1 signaling, an interesting possibility to be tested in the future.

Given the well-established role of Akt in mTORC1 activation as well as in autophagy suppression, it came as a surprise that Akt did not seem to be responsible for the hyperactivation of mTORC1 or impaired autophagy in *mdx* muscles. The unchanged Akt activity in *mdx* versus WT muscles observed in this study is consistent with other reports,^39^^,^^40^ although there are also reports of Akt hyperactivation in *mdx* muscles.^4^^,^^5^^,^^23^^,^^24^ At least some of the discrepancy may be attributed to differences in animal age or muscle type. Our experiments were conducted with young *mdx* mice, in which severe DMD phenotypes were observed. It cannot be ruled out that Akt may be involved in mTORC1 activation or autophagy impairment in dystrophic animals of different ages and/or in muscles other than TA. It is also possible that the basal activity of Akt, which is indistinguishable in WT versus *mdx* muscles and unaffected by the Akt inhibitor TCB, may be necessary for mTORC1 activation and/or autophagy suppression. Future investigations are warranted to further clarify the role of Akt in DMD muscles.

Recent years have seen a surge of discoveries in the protein synthesis–independent functions of aminoacyl-tRNA synthetases (aaRSs) in a broad range of cellular regulations.^41^, ^42^, ^43^ Each of such functions is typically unique to a specific aaRS, not shared by other aaRSs. The current work reveals LeuRS as the first aaRS to be critically linked to DMD pathology. Given its mechanism of action (via RagD), this noncanonical function of LeuRS is also unlikely to be shared with any other aaRS. This specificity would be conducive to therapeutic targeting. Indeed, the LeuRS-RagD inhibitor BC-LI-0186 is effective in restoring autophagy and muscle strength in dystrophic mice, which provides the first proof of principle for therapeutic targeting of the noncanonical function of a housekeeping protein in the potential treatment of DMD.

## Disclosure Statement

None declared.

## Supplemental Data

Supplemental Figure S1 Rapamycin inhibits S6 phosphorylation in *mdx* muscles. Wild-type (WT) and *mdx* mice at 10 weeks of age were administered rapamycin (or vehicle) intraperitoneally at 2 mg/kg body weight daily for 2 days, followed by isolation of tibialis anterior muscles and Western blot analysis. Samples from five individual mice of each genotype were loaded on the same gel, and blotted for phosphorylated S240 (pS240)/244-S6, total S6, and glyceraldehyde-3-phosphate dehydrogenase (GAPDH; loading control).

Supplemental Figure S2 BC-LI-0186 (0186) treatment does not affect differential expression of autophagy genes. Wild-type (WT) and *mdx* tibialis anterior muscles treated as in Figure 3A were analyzed for mRNA expression by quantitative RT-PCR. Data points representing individual mice are shown. Statistical analysis was performed using two-way analysis of variance, and the results are shown at the bottom of the graphs. Data are presented as means ± SEM. gen, genotype.
