# Supplementary figures and images for: Leucyl-tRNA Synthetase Contributes to Muscle Weakness through Mammalian Target of Rapamycin Complex 1 Activation and Autophagy Suppression in a Mouse Model of Duchenne Muscular Dystrophy
Source: Am J Pathol. 2024 Aug;194(8):1571–80. doi: 10.1016/j.ajpath.2024.04.006 (PMC11393824; doi:10.1016/j.ajpath.2024.04.006)

Figure S1

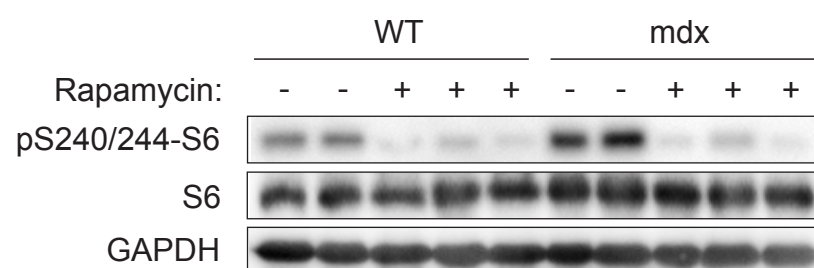

Supplement: Supplemental Figure S1 — Rapamycin inhibits S6 phosphorylation in mdx muscles. Wild-type (WT) and mdx mice at 10 weeks of age were administered rapamycin (or vehicle) intraperitoneally at 2 mg/kg body weight daily for 2 days, followed by isolation of tibialis anterior muscles and Western blot analysis. Samples from five individual mice of each genotype were loaded on the same gel, and blotted for phosphorylated S240 (pS240)/244-S6, total S6, and glyceraldehyde-3-phosphate dehydrogenase (GAPDH; loading control). [file mmc1.pdf]

Figure S2

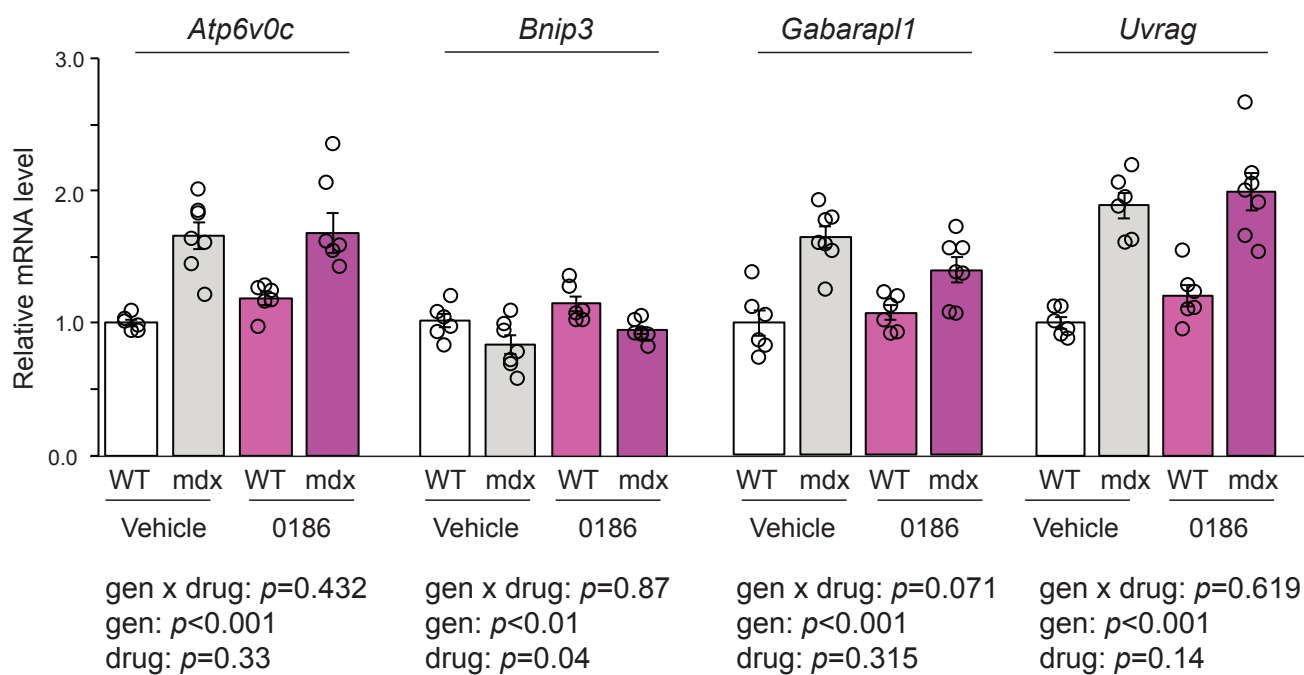

Supplement: Supplemental Figure S2 — BC-LI-0186 (0186) treatment does not affect differential expression of autophagy genes. Wild-type (WT) and mdx tibialis anterior muscles treated as in Figure 3A were analyzed for mRNA expression by quantitative RT-PCR. Data points representing individual mice are shown. Statistical analysis was performed using two-way analysis of variance, and the results are shown at the bottom of the graphs. Data are presented as means ± SEM. gen, genotype. [file mmc2.pdf]
